# B7-H4 is highly expressed in aggressive Epstein-Barr virus positive diffuse large B-cell lymphoma and inhibits apoptosis through upregulating Erk1/2 and Akt signalling pathways

**DOI:** 10.1186/s13027-019-0234-9

**Published:** 2019-08-08

**Authors:** Ying Jiang, Gangli Cai, Jun Lin, Jing Zhang, Zhilei Bo, Ying Li, Chun Wang, Yin Tong

**Affiliations:** 1 Department of Hematology, Shanghai General Hospital, Shanghai Jiao Tong University School of Medicine, 100 Haining Road, Shanghai, 200080 People’s Republic of China; 2 Department of Hematology, JinHua Hospital of TCM, 439 Shuangxi West Road, Jinhua, 321017 People’s Republic of China; 3 Department of Pathology, Shanghai General Hospital, Shanghai Jiao Tong University School of Medicine, 100 Haining Road, Shanghai, 200080 People’s Republic of China; 4 Department of Integrated Therapy, Fudan University Shanghai Cancer Center, 270 Dongan Road, Shanghai, 200032 People’s Republic of China

**Keywords:** Epstein-Barr virus positive diffuse large B cell lymphoma, B7-H4, Apoptosis

## Abstract

**Background:**

B7-H4 is among the B7 family members which may serve as a new targetable immune checkpoint molecule. It was reported that high level of serum B7-H4 level may be correlated with lymphoma. Nevertheless, the role of B7-H4 in Epstein-Barr Virus-Positive diffuse large B cell lymphoma (EBV^+^DLBCL) has not been addressed although it has been suggested that B7-H4 could promote tumor growth and metastatic progression in certain cancers.

**Methods:**

Between January 2005 and November 2017 at the department of Hematology, Shanghai Jiao Tong University School of Medicine affiliated Shanghai General Hospital 260 DLBCL samples were analyzed for EBV-encoded small RNA (EBV-EBER) by in situ hybridization. The expression level of B7-H4 in DLBCL tumor tissue was evaluated by immunohistochemistry. Furthermore, the role of B7-H4 in DLBCL was further investigated in DLBCL cell line.

**Results:**

EBV^+^DLBCL patients suffered from markedly lower overall survival (OS) and progression-free survival (PFS) rates in our study. We showed that B7-H4 was significantly overexpressed in 16 EBV^+^-subgroup cases out of 260 DLBCL patients. We further found that EBV infection in lymphoblast cells led to enhanced expression of B7-H4 followed by increased cell viability and reduced apoptosis. In contrast, inhibition of B7-H4 simultaneously impaired cell viability and induced apoptosis. Mechanistically, inhibiting B7-H4 resulted in decreased phosphorylation Erk 1/2 and Akt.

**Conclusion:**

Our study reveals a critical role of B7-H4 in EBV^+^DLBCL development by regulating cell survival and apoptosis through the Erk and Akt signalling pathways. Targetting B7-H4 may be promising in the therapy of EBV^+^DLBCL.

## Background

Epstein-Barr Virus (EBV) positive diffuse large B Cell lymphoma (EBV^+^DLBCL) is an aggressive disease with poor prognosis when treated with conventional chemotherapy [1]. New therapeutic approaches are needed for this type of lymphoma. Some research attributed the mechanisms of EBV^+^DLBCL to immunosenescence. Also, some signalling pathways were considered to be involved in the mechanisms of EBV^+^DLBCL, such as CD30, NF-kB and PD-1 [1, 2]. In recent years, immune checkpoint blockage strategies have emerged as a promising targeting therapy. PD-1 antibody shows therapeutic value in DLBCL [3]. However, still a part of patients remains resistant to these blockages. We tried to explore the other possible immune checkpoint associated with the pathogenesis of EBV^+^DLBCL.

Besides PD-1, there is another immune negative checkpoint: B7-H4. B7-H4 is among the B7 superfamily, which is shown to have inhibitory effects on T-cell activation, thereby is involved in the progress of cancers [4]. Besides its down-regulation of T-cell immunity, B7-H4 might have a direct effect on tumorigenesis independent of immunity. High expression of B7-H4 was found in various tumor tissues and related with adverse clinical course and it may serve as a target to activate anti-cancer immunity [5]. The importance of B7-H4 in the setting of lymphoma was not well studied. It was reported by Wang et al. that high level of serum B7-H4 level may be correlated with lymphoma. [6] However, the role of B7-H4 in EBV^+^DLBCL has not been addressed. Our study examined the expression of EBV in 260 DLBCL samples and tried to know the possible roles of B7-H4 in it.

## Methods

### Patients and tissue specimens

Two hundred and sixty patients diagnosed with DLBCL between January 2005 and November 2017 at the Department of Hematology, Shanghai General Hospital, Shanghai Jiao Tong University School of Medicine were analyzed. All tissues were fixed in 10% buffered formalin and embedded in paraffin. Samples were routinely stained by hematoxylin and eosin (H&E) for histological examination. The present study was approved by the Ethics Committee of Shanghai Jiao Tong University School of Medicine affiliated Shanghai General Hospital (No.2018KY258). Informed consent was obtained for experimentation with human subjects.

### Cell lines and antibodies

Pfeiffer cell line derived from human diffuse large B cell lymphoma cells were purchased from ATCC, USA. B95–8 lymphoma cells and EBV-infected marmoset white cells are a gift from Prof. Yao (The First Affiliated Hospital of Medical School of Zhejiang University). Mouse monoclonal antibody (3E8) against human B7-H4 is a gift from Institute of Infectious Diseases affiliated Zhejiang University. Goat anti-rabbit IgG antibodies and horseradish peroxidase (HRP)-conjugated goat anti-mouse IgM were purchased from Santa Cruz Biotechnology (Santa Cruz, Calif., USA). Rabbit antibodies against Bcl-2, phospho-Bcl-2, Bax, caspase 3, cleaved caspase 3, caspase 9, cleaved caspase 9, NF-κB p65, phosphor-NF-κB p65, extracellular-regulated kinase 1/2(Erk1/2), phospho-Erk1/2, Akt, phosphor-Akt and HRP-conjugated rabbit anti-D-glyceraldehyde-3-phosphate dehydrogenase (GAPDH) monoclonal antibody were purchased from Cell Signaling Technology (Danvers, Mass., USA).

### EBV- encoded small RNA ditection by in situ hybridization(ISH-EBER)

ISH-EBER analysis was performed by in situ hybridization on sections from formalin-fixed and paraffin-embedded (FFPE) tissue blocks. The assays were conducted using an ISH kit for EBER-1 (Triplex International Biosciences). Sections were stained with DAB solution and the reaction was stopped according to microscopic evaluation. The brown nuclear staining was identified as positive staining [7].

### B7-H4 immunohistochemistry(IHC)

Monoclonal antibody (3E8) against B7-H4 was prepared as previous reported [8]. IHC staining was performed using 3E8 as primary antibody followed by reagents from the EnVision System (DAKO, CA, USA). Normal mouse IgM was used as a negative control. Semi-quantitative measurements of staining intensity (0–3, least intense to most intense) and the proportion of stained cells (0–4, no stained cell stained to > 75% stained cells) were counted as previously described [8]. Positive expression of B7-H4 was defined as a combined score of equal or over 2. Overexpression of B7-H4 was defined as a combined score of equal or over 6.

### Preparation of EBV-infected Pfeiffer cell

Pfeiffer cells (1 × 10^6^ cells/ml) and the B95–8 supernatant were incubated for 2 h at 37 °C and cultured for 2 weeks with complete medium and 1 μg/ml of cyclosporine A (Sigma–Aldrich Inc.). EBV stock was extracted from an EBV-infected B95–8 marmoset cell line (a gift from Dr. Yao’s lab, The First Affiliated Hospital of Medical School of Zhejiang University). Cells were collected after being infected by EBV for 2, 6, 10 and 14 days. Cells were lysed and the expression of B7-H4 was analyzed by Western blot and flow cytometry. To inhibit B7-H4, we cultured the EBV-infected Pfeiffer cells (2 weeks, 5 × 10^5^ cells/well, 200 μl) with anti-B7-H4 antibody (5 μg/ml) at 37 °C for 3 h.

### Live cell assay

The cells were collected and seeded in triplicate in a 96-well plate. The proportion of live cells was measured by using a CellTiter 96 AQueous One Solution Cell Proliferation Assay kit (MTS; Promega) after 1 to 5 days. Absorbance at 490 nm was measured by model 680 microplate reader after cells were incubated for 4 h at 37 °C.

### Apoptosis assay

The cells were collected after 3 days of incubation with anti-B7-H4 antibody, resuspended in 100 μl of annexin V-binding buffer (10 mM HEPES/NaOH pH 7.4, 140 mM NaCl, 2.5 mM CaCl_2_) followed by stainning with FITC-conjugated annexin V (BD Pharmingen) and propidium iodide (BD Pharmingen) in dark at room temperature for 15 min. Stained cells were analyzed using a FACS Calibur (BD Pharmingen) [9].

### Western blots

The cells (1 × 10^7^ cells/sample) were washed in cold PBS and lysed in cell lysis buffer (20 mM Tris–HCl (pH 7.5), 150 mM NaCl, 1 mM Na_2_EDTA, 1 mM EGTA, 1% Triton, 2.5 mM sodium pyrophosphate, 1 mM beta-glycerophosphate, 1 mM Na_3_VO_4_, and 1 μg/ml leupeptin) (Cell Signaling Technology), and incubated on ice for 30 min. The insoluble material was removed by centrifugation at 8,000 g for 10 min. The concentration of protein was determined using BCA protein assay kit (Pierce). Lysates were separated by 12% SDS–PAGE and transferred to nitrocellulose membranes. After blocking with 5% skim milk, the blots were probed for 2 h with primary antibodies and further incubated with secondary antibodies (peroxidase-conjugated goat anti-rabbit IgG at 1:5000 or peroxidase-conjugated sheep anti-mouse IgG at 1:2000) for 1 h at room temperature. The blots were developed using an ECL kit (Amersham Pharmacia).

### Statistical analysis

Statistical analysis was performed with SPSS 23.0 software. Differences among categorical variables were evaluated by the Chi Square test. Survival distributions were estimated with the Kaplan- Meier method and groups were compared with the log-rank test. Data is judged to be statistically significant when *p* < 0.05.

## Results

### EBV^+^DLBCL patients show a more aggressive clinical course

**Fig. 1:**
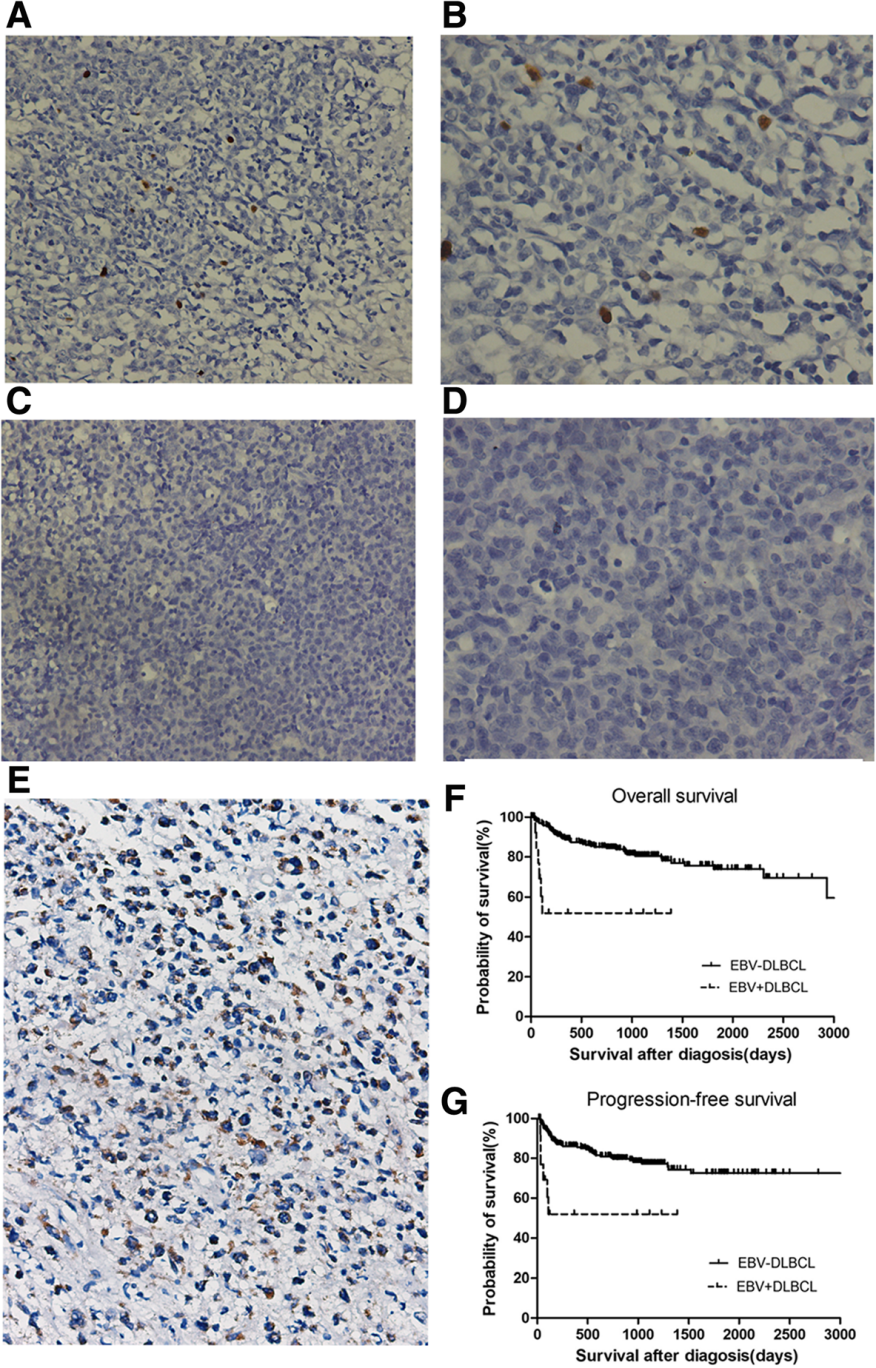
Histopathological features and survival analysis of patients with DLBCL. **a-b** ISH-EBER shows positivity with brown staining, (**a**) 20 × 40 and (**b**) 40 × 40. **c**-**d**. ISH-EBER shows negativity, (**c**) 20 × 40 and (**d**) 40 × 40. **e** Different sample from EBV^+^DLBCL patients was subjected to IHC staining using B7-H4 antibody 3E8. Normal mouse IgG was used as the negative control (*Data not shown*). Representative DLBCL sample with positive B7-H4 expression was used by Semi-quantitative measurement. Total score of this sample is 5 (intensity 3 plus proportion 2). **f**-**g** Overall survival (OS) (**f**) and progression-free survival (PFS) (**g**) EBV^+^DLBCL (*n* = 16) and EBV^−^DLBCL (*n* = 244). EBV^+^DLBCL patients had poorer prognoses than EBV^−^DLBCL patients (2 year OS rate: 51.9% vs 84.9%, *P* = 0.0001; 2 year PFS rate: 51.9% vs 80.3%, *P* = 0.0015)

To observe the prognosis of the EBV^+^DLBCL patients, we retrospectively analyzed the two hundred and sixty patients with a median age of 55 years (15 to 85 years) diagnosed with DLBCL by dividing patients into two cohorts based on the presence of EBV (EBV^+^DLBCL and EBV^─^DLBCL). We recorded the clinical features of all DLBCL patients as shown in Table 1. We performed the in-situ hybridization Epstein-Barr virus (EBV)-encoded RNA (ISH-EBER) assay on sections from these patients and found EBV^+^ tumour cells in 16 cases (6.2%) with the age ranged between 38 and 84 years (median age: 58.5 years)(Fig. 1a-d). We recorded the clinical features of all DLBCL patients as shown in Table 1. Interestingly, the overall survival (OS) rate and progression-free survival (PFS) rate were significantly lower in EBV^+^DLBCL patients than in EBV^─^DLBCL patients (2 year OS rate: 51.9% vs 84.9%, *P* = 0.0001; 2 year PFS rate: 51.9% vs 80.3%, *P* = 0.0015) (Fig. 1f-g).

**Table 1:** Clinical characteristics of patients with DLBCL

Characteristics	Total(*n* = 260)		EBV-positive(*n* = 16)		EBV-negative(*n* = 244)		*P* value
	No.	%	No.	%	No.	%	
Sex(male/female)	260(157/103)		16(11/5)		244(146/98)		0.492
Age(y) median(range)	55(15–85)		58.5(38–84)		55(15–85)		
Subgroup							
GCB	66/176	37.5	5/15	33.3	61/161	37.9	0.727
non-GCB	110/176	63.5	10/15	66.7	100/161	62.1	
Initial Chemotherapy							
R-CHOP	162/260	62.3	8/16	50.0	154/244	63.1	0.294
CHOP	69/260	26.5	4/16	25.0	65/244	26.6	0.886
other	18/260	6.9	1/16	6.3	17/244	7.0	0.913
no treatment	11/260	4.2	3/16	18.8	8/244	3.3	0.003
Therapeutic response							
CR	108/228	47.6	2/12	16.7	106/216	49.1	0.029
PR	45/228	19.8	4/12	33.3	41/216	19.0	0.224
SD	4/228	1.8	0/12	0.0	4/216	1.9	0.634
PD	71/228	31.3	6/12	50.0	65/216	30.1	0.147
Clinical stage							
I	32/251	12.7	1/15	6.7	31/236	13.1	0.466
II	39/251	15.5	1/15	6.7	38/236	16.1	0.328
III	58/251	23.1	0/15	0	58/236	24.6	0.029
IV	122/251	48.6	13/15	86.7	109/236	46.2	0.002
B symptoms present	95/260	36.5	10/16	62.5	85/244	34.8	0.026

*CR* Complete remission, *PR* Partial remisssion, *SD* Stable disease, *PD* Progression of disease, *R* Rituximab

*P* values are for the comparison of EBV^+^ and EBV^─^DLBCL patients

### Significantly enhanced expression of B7-H4 in EBV^+^DLBCL patients

To determine the expression of B7-H4 in EBV^+^ and EBV^─^ cohorts, we used IHC staining of B7-H4 in 16 tissue sections from EBV^+^DLBCL patients and 155 tissue sections from EBV^─^DLBCL patients. We observed that 13 (81.3%) out of 16 tissue sections from EBV^+^DLBCL patients and 121 (78%) out of 155 tissue sections from EBV^─^DLBCL patients stained positive for B7-H4 (*P* = 0.768) (Fig. 1e). However, B7-H4 was markedly more overexpressed in EBV^+^DLBCL (54%, 7/13) than that in EBV^─^DLBCL (7%, 11/155) as scored by staining intensity and proportion of positively stained cells (*P* = 0.000) (Table 2).

**Table 2:** The expression of B7-H4 in EBV^+^ and EBV^─^DLBCL patients

Group	EBV^+^DLBCL	EBV^−^DLBCL	*P* value
No. of B7-H4 expression			
Negative	3(18.75%)	34(21.94%)	0.768
Positive	13(81.25%)	121(78.06%)	
Overexpression	7(43.75%)	11(7.09%)	0.000
Case no.	16	155	

### Inhibition of B7-H4 led to decreased cell viability and enhanced apoptosis in EBV-infected Pfeiffer cells

**Fig. 2:**
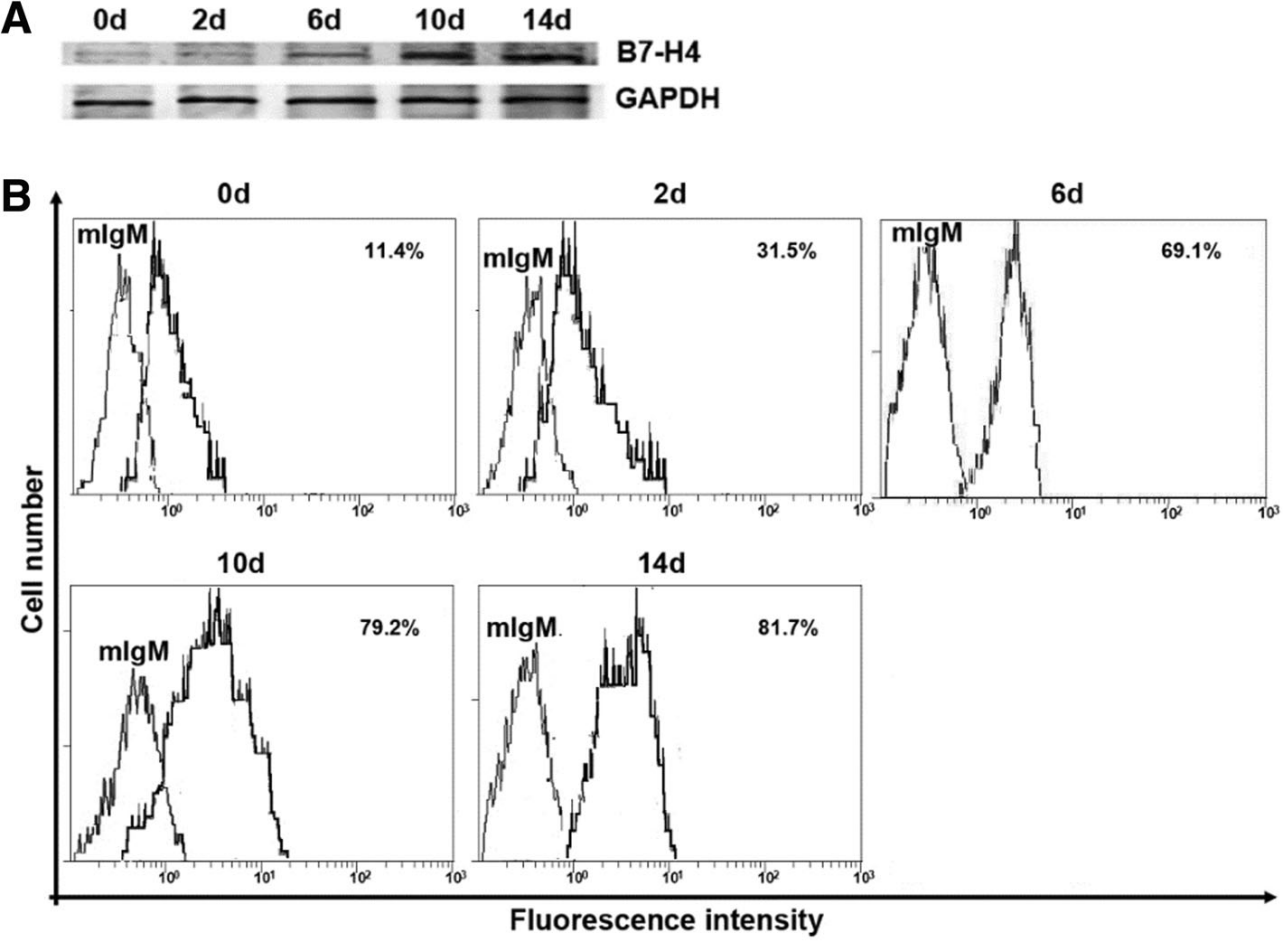
B7-H4 expression on EBV-infected Pfeiffer cells. Pfeiffer cells were infected with EBV for 0,2,6,10,14 days. Cells were collected from each time point for analysis. **a** Cellular proteins were analyzed by Western blotting analysis with 3E8 anti-B7-H4 monoclonal antibodies. **b** Flow cytometry analysis with 3E8 mAb. Normal mouse IgM was used as a negative control. Cells were resuspended in a solution of FITC-goat anti-mouse IgM. Finally, cells were analyzed using a FACScan flow cytometer

**Fig. 3:**
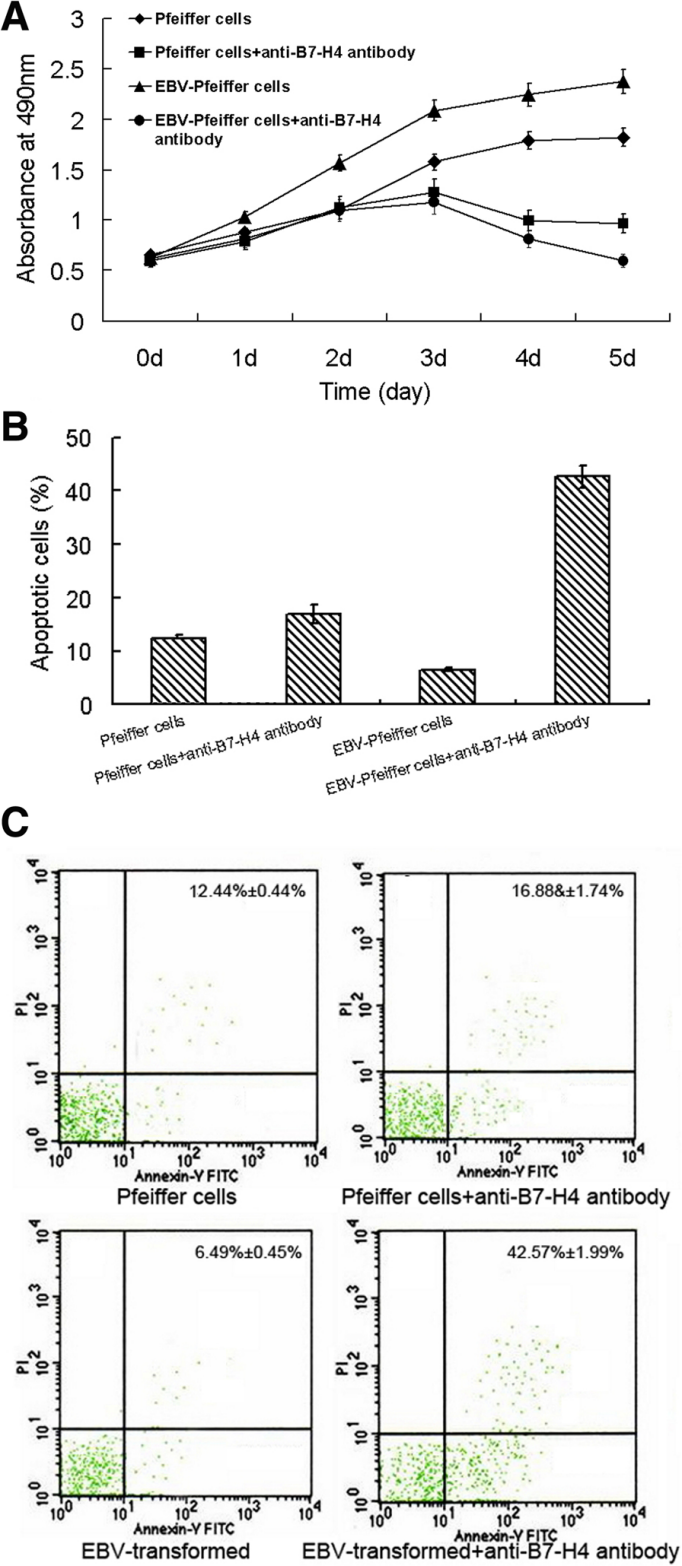
Anti-B7-H4 monoclonal antibody reduces live cells and induces apoptosis of EBV-infected Pfeiffer cells. Pfeiffer cells were transfected with EBV and were cultured with anti-B7-H4 antibody (5 μg/ml) for 3 days. **a** Live cells were counted by MTS assay. **b** Cells stained with FITC-annexin V and propidium iodide (PI) were analyzed by flow cytometry. **c** Representative of cytofluorimetric analysis

We further investigated whether EBV infection causes B7-H4 overexpression in Pfeiffer cells which expresses B7-H4. We found that B7-H4 was lowly expressed on surface of Pfeiffer cells without EBV infection, but gradually increased at day 2, 6 and 10 after infection of EBV (Fig. 2). This result is consistent with our observation in EBV^+^DLBCL patients. Moreover, we detected substantially increased live cells and declined apoptosis in EBV-infected Pfeiffer cells (Fig. 3a-b). Importantly, when treated with anti-B7-H4 antibody which inhibits the expression of B7-H4, EBV-infected Pfeiffer cells dropped and the apoptosis was increased (Fig. 3b-c).

### Inhibition of B7-H4 led to apoptosis of EBV-infected Pfeiffer cells via caspase activation

**Fig. 4:**
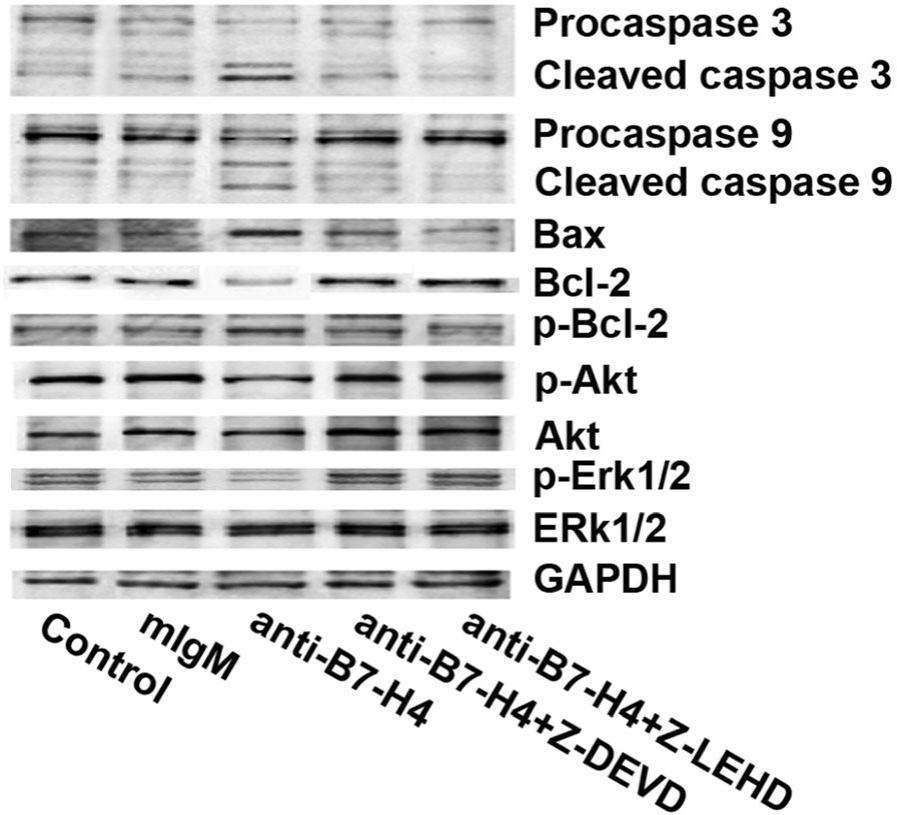
Anti-B7-H4 antibody upregulates apoptotic proteins and downregulates mitogenic signaling. EBV-infected Pfeiffer cells were cultured with or without the Z-DEVD-fmk (caspase 3 inhibitor) or Z-LEHD-fmk (caspase 9 inhibitor) for 2 h before stimulation with anti-B7-H4 antibodies (5 μg/ml) for 3 days. Cellular proteins were observed by Western blotting after 72 h with antibodies of Bcl-2, p-Bcl-2, Bax, caspase 3, caspase 9, p-Akt, Akt, p-Erk1/2, Erk1/2 and GAPDH

To explore the mechanism through which B7-H4 inhibited apoptosis, we analyzed caspase 3 and caspase 9 by Western blot in EBV-infected Pfeiffer cells. We noticed that inhibition of B7-H4 increased the expression of cleaved caspase 3 and cleaved caspase 9. When cells were treated with caspase 3 inhibitor (Z-DEVD-fmk) and caspase 9 inhibitor (Z-LEHD-fmk) before incubation with anti-B7-H4 antibodies, expression of cleaved caspase 3 and cleaved caspase 9 significantly decreased compared with in EBV-infected Pfeiffer cells treated with anti-B7-H4 antibody only (Fig. 4). We next analyzed whether Bax and Bcl-2 plays a role in B7-H4 mediated inhibition of apoptosis. As shown in Fig. 4, the pro-apoptotic protein Bax was increased by anti-B7-H4 treatment, while the anti-apoptotic protein Bcl-2 was decreased after B7-H4 inhibition. These data suggest that in addition to caspase 3 and 9, B7-H4 suppressed apoptosis through altering the Bcl-2/Bax ratio. Furthermore, we found that B7-H4 directly regulated the activation of Erk1/2 and Akt by Western blot analysis. As shown in Fig. 4, inhibition of B7-H4 reduced the phosphorylation of Erk1/2 and Akt.

## Discussion

Several studies reported that EBV^+^DLBCL patients had worse prognosis when compared to EBV^─^DLBCL patients (5 year OS rates of 25% in EBV^+^DLBCL and 65% in EBV^─^DLBCL) [1, 10]. Morales et al. found that 11 cases (15%) positive for ISH-EBER were associated with poor prognosis. The median OS in EBV^+^DLBCL was 7 months compared with 47 months in EBV^─^DLBCL [11]. Similar findings were reported in other studies [12, 13]. In line with these data, we also found the worse outcome of EBV^+^DLBCL patients compared with EBV^─^DLBCL patients (2 year OS rate: 51.9% vs 84.9%, *P* = 0.0001; 2 year PFS rate: 51.9% vs 80.3%, *P* = 0.0015, Fig. 1f-g).

T-cell mediated anti-tumor immunity plays an important role in preventing tumorigenesis. The classic B7 family provides costimulatory signals to regulate T-cell response. Blocking the negative costimulatory signals such as PD-1 improved immune response in lymphoma [14]. Besides PD-1, B7-H4 has been raised importance as another targetable checkpoint inhibitor. B7-H4 is a member the B7 superfamily, which was highly expressed across a wide variety of malignancies and was correlated with tumor aggressiveness via inhibiting T-cell activation [5, 15–17]. Recently, 13 types of cancers including melanoma, prostate, stomach, lung, ovary, cervix, kidney, colon, breast, liver and pancreas cancer, were reported to correlate with B7-H4 in retrospective analyses [18–23]. Furthermore, B7-H4 might regulate tumorigenesis beside its function immunity. Increased B7-H4 expression in tumor cells was correlated with decreased cell apoptosis and enhanced growth of tumor cells. Thus, B7-H4 may serve as an effective biomarker for cancer diagnosis and prognosis [24]. The role of B7-H4 is unclear in DLBCL, which is the most common type of Non-Hodgkin’s lymphoma (NHL). In our study, we found for the first time that the expression of B7-H4 was largely enriched in EBV^+^DLBCL compared with that in EBV^─^DLBCL, suggesting a critical role of B7-H4 in the development of EBV^+^DLBCL. These results indicate the B7-H4 might serve as a critical mediator the development of EBV^+^DLBCL.

Previously, Song et al. reported enhanced expression of B7-H4 induced apoptosis in EBV^+^ B cells [9]. Interestingly, EBV-infected cells were able to escape immune surveillance via B7-H4 [25]. These data implies that B7-H4 may inhibit the proliferation and induce apoptosis of B cells to weaken the production of immunoglobulin, thus suppresses adaptive immunity. Song et al. further reported that B7-H4 significantly reduced cell growth via downregulating CDK4/6, CDK2, cyclin E and cyclin D, and enhanced apoptosis in lymphoma cell lines [26]. Conversely, Qian et al. showed that B7-H4 activation enhanced oncogenicity and inhibited apoptosis in pancreatic cancer cells [27]. The studies add the complexity of the role of B7-H4 in oncogenesis. We further elucidated the relationship between EBV and B7-H4 by infecting the DLBCL cell line Pfeiffer cells with EBV. We found that the expression of B7-H4 was upregulated in EBV-infected Pfeiffer cells, which increased live cells and reduced apoptosis. These in vitro results indicate that B7-H4 may contribute to transformation of lymphoma by EBV infection in vivo.

To determine the molecular mechanism underlying the function B7-H4 in EBV-infected cells, we utilized the anti-B7-H4 monoclonal antibody (3E8) which inhibited B7-H4 [28]. We found that B7-H4 by increased cell viability and inhibited apoptosis, suggesting an enhanced oncogenicity of lymphoma cells. Our results are consistent with previous studies. For example, Cheng et al. showed that overexpression of B7-H4 promoted tumorigenesis of ovarian cancer in immunodeficient mice by increasing proliferation, cell adhesion, migration, and invasion of cancer cells [29]. Knockdown of B7-H4 in a breast cancer cell line increased caspase activity and apoptosis [30]. B7-H4 also enhanced tumor growth and inhibited apoptosis in pancreatic cancer [27]. Here, we showed that B7-H4 contributed to the surviving of DLBCL cells. It’s worth mentioning that our results disagree with the study by Park et al. which showed cell cycle arrest and apoptosis in B7-H4^+^ lymphoma cell lines (Raji and IM-9 cells). The discrepancy is likely to be a result of different lymphoma cell lines and anti-B7-H4 antibody used. In our study, we used Pfeiffer cell for study because this cell line is one of DLBCL cell lines without the expression of B7-H1. Otherwise, it may confuse our investigation of B7-H4.

Many anti-cancer drugs aim at inducing apoptosis [31]. The increased Bax/Bcl-2 ratio is likely to be followed by cytochrome C release, caspases 9, 3, 6, and 7 activation, which eventually lead to apoptosis [32]. Also Erk1/2 and PI3K/Akt pathway is a critical regulator of apoptosis by directly phosphorylating and inactivating caspase 3, 6, 8, and 9 [33, 34]. Park et al. reported that B7-H4 modulated the cell cycle through downregulating of the Akt in EBV^+^ B-cell lymphoma [26]. Indeed, our data showed that caspases 9 and 3 were activated and the Bax/Bcl-2 ratio was altered after inhibition of B7-H4 in EBV-infected Pfeiffer cells caused apoptosis. In our study, inhibition of B7-H4 by anti-B7-H4 antibody also reduced the protein level of phospho-Erk1/2 and phospho-Akt, suggesting that B7-H4 is responsible for inhibition of apoptosis via upregulating the Erk1/2 and Akt signalling pathway. Taken together, these findings demonstrate that B7-H4 effectively inhibit apoptosis via multiple pathways including caspases 3 and 9, Bcl-2 and Bax as well as Erk1/2 and Akt signalling pathways.

## Conclusion

Our study demonstrated that B7-H4 was highly expressed in EBV^+^DLBCL which showed a more aggressive clinical course than EBV^─^DLBCL. Consistently, the infection of DLBCL cell line by EBV increased the expression of B7-H4. B7-H4 promoted cell viability by inhibiting apoptosis in EBV-infected DLBCL cell line via Erk1/2 and Akt pathway. Thus, we revealed a critical role of B7-H4 may in development of EBV^+^DLBCL. These data shed light on B7-H4 as a potential therapeutic target in EBV^+^DLBCL patients. Future studies are required to test the function of B7-H4 in vivo. Besides, due to the rarity of EBV^+^DLBCL patients, collaborative studies among multiple institutions are warranted.

## Data Availability

Not applicable.

## Abbreviations

DLBCL: Diffuse large B cell lymphoma
EBV: Epstein-Barr Virus
EBV^+^DLBCL: Epstein-Barr Virus-Positive diffuse large B cell lymphoma
EBV^─^DLBCL: Epstein-Barr Virus negative diffuse large B cell lymphoma
Erk1/2: Extracellular-regulated kinase 1/2
FFPE: Formalin-fixed and paraffin-embedded
H&E: Hematoxylin and eosin
HRP: Horseradish peroxidase
ISH-EBER: EBV-encoded small RNA with in situ hybridization
OS: Overall survival
PFS: Progression-free survival
